# Notes and News

**Published:** 1895-07-06

**Authors:** 


### Notes and News.

(Collected and Communicated.)

HOSPITAL MEETINGS, &c.

London Homœpathic Hospital, Great Ormond Street.—Opening of new building by H.R.H. Princess Mary Adelaide, Duchess of Teck, on Tuesday, July 9th, at four o'clock.

A Festival in aid of the London Homœopathic Hospital will be held on July 10th at the Hôtel Métropole.

The Laying of the Foundation Stone of the new School Buildings of the Royal Medical College, by the Prince of Wales, will take place on Monday, July 8th, not on Tuesday, the 9th inst., as previously announced.

The distribution of scholarships, medals, prizes, and certificates to the students at Guy's Hospital by the Master of the Salters' Company was made the occasion of a large gathering on July 3rd. The huge tent presented a gay and picturesque appearance thronged with guests, and the weather added much to the enjoyment of the extensive grounds. The wards, museum, and college were open to visitors, and much interest was taken in the buildings which comprise the dental department.

Professor W. William Williamson, of, and for over forty years professor at, Owens College and an eminent man of science, died at Clapham last week, aged seventy-eight. His first studies tended towards natural history, but he gave up his post at the Manchester Natural History Museum to study medicine at University College. He acquired a large private practice at Manchester, being connected with several medical institutions also. He continued his studies, and his valuable contributions to natural science rendered him famous. He obtained the Royal Society's Gold Medal in 1874. He was on the professoriate of Owens College from its foundation, occupying various chairs from 1851 to 1892, when he resigned office. His remarkable collection of fossil plants is preserved in the museum of the Owens College. He received numerous marks of distinction during his career, both at home and abroad.

It is announced that the subject of the Essays for the Howard Medal of the Royal Statistical Society, which will be awarded in 1896 with £20 as heretofore, is as follows: "School Hygiene, in its Mental, Moral, and Physical Aspects." The essays should be sent in on or before June 30th, 1896.

A very large and brilliant conversazione was held at University College on Thursday, June 27th. The programme included some excellent music, and a large number of interesting exhibits, amongst the latter being the spectra of argon and helium, which attracted a number of spectators. The whole building was beautifully decorated with flowers and arranged by the lady students. Some of the chief rose-growers sent collections of roses, which added to the attractions of a most enjoyable evening.

We believe that the suggestion to amalgamate King's College and Charing Cross Hospitals has been under consideration by some of those immediately concerned. It appears that the constitution of King's College Hospital is so opposed to anything of the kind as to render it impracticable. The hospital is governed by King's College, which is a Church of England institution, and an obstacle is thus presented to the scheme of amalgamation. There can be no doubt that it would be to the advantage of everybody concerned if the King's College and Charing Cross Hospitals could be amalgamated. Failing this, we hope that the authorities of Charing Cross Hospital will very carefully consider what is necessary to enable them to secure the whole of the triangular site upon a portion of which the present building stands. We are convinced that a bold scheme which would provide for the acquirement of the whole site by the hospital authorities, and the erection thereon of a modern hospital of the highest excellence, would command public sympathy, and secure the subscription of the necessary funds.

The Committee of Management of the Royal Free Hospital announce that their Royal Highnesses the Prince and Princess of Wales have graciously intimated their intention of honouring the hospital by a visit on Monday, July 22nd, at one o'clock, for the purpose of opening the new front building. The cost of the new front building, together with a laundry, the reconstruction of the drainage system, fire extinguishing appliances, and other necessary improvements, has been about £30,000, towards which sum the committee have obtained £24,000, thus leaving a balance of £6,000 still to be raised. In order that this sum of £6,000 may be raised by the opening day, the committee are obliged to again appeal to the governors and friends of the hospital. If each governor, including those who have already responded to the previous appeal on behalf of the building fund, would now contribute or obtain an additional gift, the balance required would soon be realised, and the hospital be completed free from debt. Her Royal Highness the Princess of Wales has consented to receive from ladies and children purses containing not less than £5 5s., and the secretary will duly supply purses and collecting cards to those who apply for them.

